# True versus False Parasite Interactions: A Robust Method to Take Risk Factors into Account and Its Application to Feline Viruses

**DOI:** 10.1371/journal.pone.0029618

**Published:** 2012-01-03

**Authors:** Eléonore Hellard, Dominique Pontier, Frank Sauvage, Hervé Poulet, David Fouchet

**Affiliations:** 1 Université de Lyon, Lyon, France; 2 Université Lyon 1, CNRS, UMR5558, Laboratoire de Biométrie et Biologie Evolutive, Villeurbanne, France; 3 Merial, Recherche et Développement, Lyon, France; Albert Einstein College of Medicine, United States of America

## Abstract

**Background:**

Multiple infections are common in natural host populations and interspecific parasite interactions are therefore likely within a host individual. As they may seriously impact the circulation of certain parasites and the emergence and management of infectious diseases, their study is essential. In the field, detecting parasite interactions is rendered difficult by the fact that a large number of co-infected individuals may also be observed when two parasites share common risk factors. To correct for these “false interactions”, methods accounting for parasite risk factors must be used.

**Methodology/Principal Findings:**

In the present paper we propose such a method for presence-absence data (i.e., serology). Our method enables the calculation of the expected frequencies of single and double infected individuals under the independence hypothesis, before comparing them to the observed ones using the chi-square statistic. The method is termed “the corrected chi-square.” Its robustness was compared to a pre-existing method based on logistic regression and the corrected chi-square proved to be much more robust for small sample sizes. Since the logistic regression approach is easier to implement, we propose as a rule of thumb to use the latter when the ratio between the sample size and the number of parameters is above ten. Applied to serological data for four viruses infecting cats, the approach revealed pairwise interactions between the Feline Herpesvirus, Parvovirus and Calicivirus, whereas the infection by FIV, the feline equivalent of HIV, did not modify the risk of infection by any of these viruses.

**Conclusions/Significance:**

This work therefore points out possible interactions that can be further investigated in experimental conditions and, by providing a user-friendly R program and a tutorial example, offers new opportunities for animal and human epidemiologists to detect interactions of interest in the field, a crucial step in the challenge of multiple infections.

## Introduction

Numerous parasites species circulate simultaneously in natural populations. Many of them are able to infect a same host species and a host individual can therefore be infected by several parasites at the same time. These multiple infections are not only common in nature but usually more frequently encountered than infections by a single parasite [1]. Within a host individual, parasites can thus interact, either in a synergistic manner (parasite A favours infection by parasite B or worsens the symptoms caused by B) or in an antagonistic manner (parasite A decreases the infection risk by parasite B or reduces the symptoms caused by B) [2]. As these interactions can have important epidemiological, biological and clinical consequences (e.g., [3]–[7]), detecting, understanding and evaluating them is essential to understand the phenomena and to control and manage infectious diseases.

In recent years, the question of polyparasitism has attracted considerable attention [4], [8], [9], although in reality the subject has a long history of experimental investigation under laboratory conditions [10], [11]. Many epidemiological studies have also been conducted on the main human pathogens, motivation for the study of polyparasitism being in particular driven by the urgency to understand the epidemiological and clinical consequences of infection by parasites potentially interacting with HIV and other emerging diseases [12] and the mechanisms of their interactions. A large amount of work indeed revealed interactions between HIV and tuberculosis, malaria, sexually transmitted diseases, and helminths (e.g., [6], [13]–[20]); as well as interactions between plasmodia parasites and helminths (e.g., [21]–[24]). Studies on animal hosts also revealed interactions between their parasites, with many studies on helminth communities (mammals: [4], [25], [26], birds: [27], fish: [28]), and fewer on protozoan species (e.g., [29]) or viruses (e.g., [30]). Many diseases have been revealed to be affected by the presence of other disease-causing agents, altering the rates of species co-occurrence, levels of infection and disease severity. Parasite interactions have also been shown to affect the success of parasite vaccination strategies [31] and could be involved in disease (re)emergence [32], reinforcing the interest of these studies.

If laboratory experiments have clearly demonstrated that interspecific parasite interactions occur, often mediated by host immune responses [1], [33]–[35], attempts to detect such effects in natural populations have generally been less successful. Indeed, detecting their existence on the field is not easy, due to complex networks of indirect effects making it difficult to infer underlying processes. Field studies are however essential as experimental systems are oversimplified and require an existing suspicion of interaction between the studied parasites. In addition, only studies in natural populations can give access to infection and co-infection probabilities. In other words, before studying their mechanisms in the lab, interactions of interest must be identified in the field. Main difficulties encountered in field studies are methodological. Many confounding factors can create statistical associations between parasites even if there is no true biological interaction between them, which may alter conclusions about the importance of interspecific interactions [36]–[40]. A similar transmission mode, for example, can alone increase the risk of co-infection. The excess of positive associations found in strongylid communities in domestic horses, ruminants and macropod marsupials is in particular likely to be due to the common habit of these hosts feeding on pastures contaminated with the larvae of a number of nematode species [41]–[43]. In addition, environmental, behavioural or host-specific factors can be associated with both types of infection and influence epidemiological and geographic patterns of infection and disease. Among such common risk factors, some have long been recognised, such as sexual behaviours for sexually transmitted diseases (e.g., [44]), socio-economic status for infections particularly prevalent in poor regions such as helminth infection and malaria [45], or age for many diseases (e.g., [46]). As apparent associations between two infections may be due to common risk factors, they are crucial to identify and to take into account in the analysis. However, such confounding factors are difficult to control and few methods enable to take them into account.

A variety of analytical approaches have been suggested to detect associations in parasite communities, primarily focusing on macroparasite (parasitic helminth) communities (e.g., [4], [39], [47], [48]). However, they implicitly assume that the direction and strength of an observed association between parasite species reflects an underlying biological interaction, and their reliability to detect interactions has been recently questioned [49]. The adoption of a generalized linear mixed modelling (GLMM)-based approach has been rather suggested by Fenton et al. [49] (see also [50]). Apparently more robust to detect interactions between macroparasites, this method has the advantage of offering the opportunity of taking into account the variance caused by other factors.

Nevertheless, field data, particularly relating to microparasites, are most of the time serological (i.e. presence-absence data). Indeed, viral excretion is usually too short to make antigen detection an efficient tool to follow microparasites in natural populations, as host capture and sampling would have to be done exactly during the excretion period, especially during non-epidemic phases. Most field data are thus limited to observed frequencies of seronegative, seropositive and doubly seropositive individuals. In this context, the search for potential interactions between pairs of microparasites is traditionally done by calculating odds ratios in stratified data or by a Pearson's chi-square test of independence (e.g., [29], [51], [52]). The latter compares the observed frequencies to the frequencies expected if parasites are independent, under the null hypothesis that the joint distribution of the cell counts in a 2-dimensional contingency table is the product of the row and column marginals. However, such methods ignore confounding factors and/or the possible simultaneous action or interaction of several of them. Significant associations detected in this manner can therefore be either true biological interactions or statistical associations, with no means of distinguishing the two. Alternative methods have been therefore proposed to determine the expected frequencies in a modified chi-square analysis. Some are based on the estimation of “pre-interactive” species prevalences [53], which requires previous knowledge of dominance relationships between parasites species. Some others are based on log-linear models [e.g.], [ 54]–[56]. In addition, another way to take risk factors into account is to include them in a logistic regression analysis and to determine whether parasite B status is still a predictor of parasite A status [52], [57]. However, the main drawback of methods based on log-linear or logistic regression models is that they are based on an asymptotic approximation of the deviance, which might not be relevant for small sample size data.

In the present paper, we propose another method (termed the “corrected chi-square”) to detect microparasite interactions from serological data, based on an adaptation of the Pearson's chi-square test. By combining logistic regressions and chi-square tests, we are able to calculate the expected frequencies of co-infected individuals if parasites are independent considering their risk factors, and to compare them to the observed ones. In a first step, we perform a theoretical comparison of the robustness of the corrected chi-square and the logistic regression approaches. In a second step, both approaches are applied to serological data obtained in natural populations of domestic cats to search for potential interactions between four feline viruses. The domestic cat is indeed an appropriate model to investigate such questions as its main viruses are well known and rather easy to survey on the field, and its natural populations, although very flexible in their social and spatial organisation, have been extensively studied [32], [58]–[62].

## Materials and Methods

### 1. Statistical analysis

#### 1.1. Logistic regression analysis

A first way to test the interaction between two pathogens is to test the effect of the serological status to one virus on the probability of being seropositive to the other. A logistic regression was used for that purpose. The approach allows correcting for common risk factors by adding known or suspected risk factors as correction variables. The logistic regression model reads:
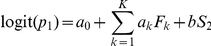
Where *F_k_* denotes the *k*-th risk factor, *p_1_* is the probability of seropositivity to pathogen 1 and *S_2_* the serological status to pathogen 2. The coefficients *a_k_* (*k* = 0…*K*) and *b* are the coefficients of the logistic regression.

The interaction between the two pathogens was tested using a likelihood-ratio test (*LRT*) testing H0: *b* = 0 vs H1: 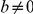. The asymptotic chi-square approximation was used to derive the P-value of the test of independence between the two viruses [63].

#### 1.2. Corrected Pearson's chi-square tests

The corrected chi-square approach is based on the idea that the coefficients of the logistic regression of the two viruses can be used to estimate the number of seronegative, single- and double-seropositive individuals expected if the two pathogens are independent. As the classical chi-square, the corrected chi-square compares the observed (*O_i,j_*) and theoretical (*E_i,j_*) numbers of individuals with different combinations of status (seropositive or seronegative) for the two pathogens using the chi-square statistic:
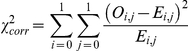
where *i* is the status to pathogen 1 (0 for seronegative and 1 for seropositive) and *j* is the status to pathogen 2. To calculate the *E_i,j_*, for each pathogen taken separately, a logistic regression including *K* risk factors (see previous section) is run to estimate the probability of being seropositive for each individual (termed 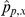 for individual *x* and pathogen *p*, 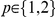):
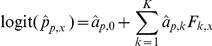
Where 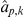 denotes the estimation of the regression coefficients for pathogen *p* and *F_k,x_* the value of the *k*-th risk factor in individual *x*. The theoretical contingency table is then deduced from these probabilities:
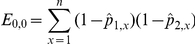


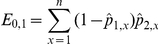


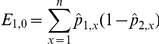


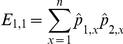



For each pair of viruses, the distribution of the corrected chi-square was determined by a parametric bootstrap run as follows:

*Step 1:* Estimated seropositivity probabilities (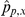) are used to generate *in silico* serological data for both pathogens independently.
*Step 2:* The corrected chi-square is calculated for this *in silico* dataset.

Steps 1 and 2 were repeated 1000 times, leading to 1000 independent realisations of the corrected chi-square statistic under the null hypothesis of independence between the two pathogens.

Two ways of calculating the P-value were derived from this procedure. P-value1 was estimated assuming that the corrected chi-square is proportional to a chi-square with one degree of freedom, the coefficient of over- (or under-) dispersion (ĉ) being defined by the mean of the bootstrapped corrected chi-square. P-value2 was given by the proportion of bootstrapped corrected chi-squares which were smaller than the observed value. In principle, P-value2 is better (no assumption on the distribution of the Likelihood Ratio Test, LRT, is made), but requires running enough simulations, which may be long in some cases. P-value1 allows working with smaller numbers of simulations when simulation times are too long.

The R program is available as supplementary file (File S2) and can be applied to any presence-absence data to calculate the corrected chi-square and the associated P-values. A tutorial example (File S3) illustrates its use step-by-step using an example dataset (File S4).

### 2. Robustness of the two approaches

The main criticism that could be made to the logistic regression approach is that it is based on the asymptotic distribution of the LRT. In practice, the chi-square approximation is true only for large datasets. In the present paper we investigated the robustness of the logistic regression to different sample sizes and numbers of correction risk factors. We also aimed to compare how robustness is affected by the type of risk factors considered (qualitative or quantitative). The same investigations were performed with the corrected chi-square test to compare the robustness of the two approaches.

For that purpose, random seroprevalence datasets were generated, assuming independent viruses. Random data were always generated assuming that all individuals had an independent 0.5 probability of being seropositive for each pathogen. *N_F_* randomly generated risk factors were considered in the logistic regression for the two pathogens. By construction these factors have no effect (they are chosen independently of the serological status of the individuals) but from a theoretical point of view it is interesting to measure how their inclusion in the model can introduce biases depending on the approach.

Randomly generated factors could be either qualitative or quantitative. For simplicity, qualitative factors had only two modalities, individuals having a 0.5 probability of being in each one. Quantitative factors were chosen for each individual randomly according to a standard normal distribution. To investigate how the nature of risk factors affects robustness, three scenarios were tested: i) all factors are qualitative; ii) all factors are quantitative and iii) half of the factors are quantitative while the other half are qualitative factors (mixed scenario).

Our objective now was to understand how data characteristics (the number of individuals, *n*, the number of factors, *N_F_* and their type, scenario i, ii or iii) would affect the probability of wrongly concluding that there is an interaction between the two pathogens (type I error). For a given combination of these characteristics, a thousand random seroprevalence datasets were generated and we estimated the type I error associated to each approach as the proportion of random datasets for which the P-value was below 5%.

### 3. Application to cat data

#### 3.1. Ethics Statement

The field work has been made by qualified people according to the French legislation. Accreditation has been granted to the UMR-CNRS 5558 (accreditation number 692660703) for the program.

#### 3.2. The feline viruses

The Feline Immunodeficiency Virus (FIV), is a major non-traumatic cause of death in adult cats, and is associated with immunosuppression causing secondary infections [64]. This retrovirus can infect other felids, most of which are threatened or endangered species e.g., the European wildcat (*F. s. silvestris*) [64]–[66]. It is mainly transmitted by bites, through a direct horizontal mode [67], principally during aggressive or sexual contacts [64], [68]. The Feline Herpesvirus (FHV) and the Feline Calicivirus (FCV) are responsible of upper respiratory tract disease, of concern in veterinary medicine [69], [70]. Both viruses are transmitted through ‘amicable’ contacts, by oral, nasal and ocular secretions during close interactions [71], [72]. FHV infected cats become asymptomatic carriers, but the latent infection can be reactivated by a stress (i.e., change of habitat, lactation or fights between males; [73]). The Feline Parvovirus (FPV) infects all felids, as well as other carnivores [74], and FPV infection may be fatal especially in kittens [75]. The virus is transiently excreted in feces, urine, saliva and vomiting and its high resistance in the environment (still infectious after 13 months at 4–25°C; [76]) makes indirect transmission through feces and contaminated areas largely predominant [77], [78].

#### 3.3. Serological data

The serological statuses for FIV, FHV, FCV and FPV were obtained in 2007 in 15 natural rural populations of domestic cats in North-Eastern France [62], [79]. Cats were captured using baited traps or directly caught by the owner, anaesthetized, measured, and blood samples were taken from the jugular vein. FIV-antibodies were immediately searched for with a commercial kit using the ELISA method (SNAP Combo +, Idexx), whereas specific antibodies against FHV, FCV or FPV were measured by a specific blocking ELISA [80]. None of the cats was vaccinated. All six pairs of viruses were tested for potential association. Between 467 and 474 cats were tested for each virus and 465 to 469 were double-tested (depending on the virus pair).

Previous analyses using logistic regression models with the same dataset revealed the combination of risk factors that were supported by our data [62]. Five factors were initially investigated: age (AGE), sex (SEX), way of life (owned or unowned, WOL), orange phenotype (orange or non orange, PHENO) and body mass (MASS) and one correction factor (the population of origin, POP) was considered. For each virus, the most appropriate model was selected using the Akaike Information Criterion adjusted for small sample size (AICc, [81]). Ideally, all factors potentially creating apparent associations should be included in the model. But to limit the number of correction risk factors, the minimal model containing the identified risk factors for the two viruses was retained as a compromise for each pair (Table 1).

**Table 1:** Risk factors models used to test for potential association between pairs of feline viruses.

Viruses	Model
FIV-FHV	POP+AGE*WOL*SEX+MASS
FIV-FCV	POP+AGE*WOL*SEX+AGE*WOL*PHENO+MASS
FIV-FPV	POP+AGE*PHENO+AGE*WOL*SEX
FHV-FCV	POP+AGE*WOL*PHENO+MASS
FHV-FPV	POP+AGE*WOL+AGE*PHENO+MASS
FCV-FPV	POP+AGE*WOL*PHENO+MASS

## Results

### 1. Robustness of the two approaches

**Figure 1:**
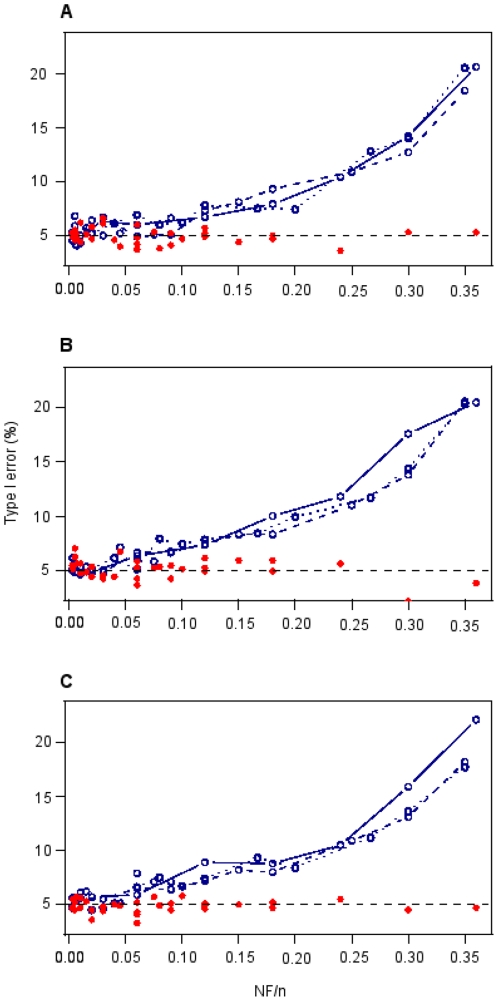
Robustness of the two approaches. Type I error (%) for the logistic regressions approach (blue empty points) and the corrected chi-square test (red full points) depending on the ratio of the number of factors to the sample size (N_F_/n), considering three scenarios: i) all factors are qualitative (A); ii) all factors are quantitative (B) and iii) half of the factors are quantitative and the other half are qualitative (C). The type I error of the corrected chi-square tests represented here is based on P-value2 but similar results were observed with P-value1 (Fig. S3). Note that for the logistic regression approach, points resulting from a given sample size were linked to see the effect of the N_F_/n ratio for different sample sizes (solid line: n = 100, dashed line: n = 200, dotted line: n = 300). The dashed horizontal line represents a type I error of 5%.

The corrected chi-square was robust for all tested sample sizes and numbers of parameters, whatever the nature of the factors (scenarios i, ii, iii) and the method used to calculate the P-value (P-value1, P-value2, see File S1 and Fig. S3 for more details). The type I error of this method remained indeed very close to 5% (Fig. 1). On the contrary, the robustness of the logistic regression approach decreased with the N_F_/n ratio (number of factors/sample size). In scenarios i (only qualitative factors) and ii (only quantitative factors), the type I error was around 5% for a ratio of 0.005, around 8% for a ratio of 0.15 and around 20% for a ratio of 0.35. It became significantly different from 5% for ratios larger than 0.12 (type I error = 6.7%, z = 2.47, p = 0.019) and 0.08 (type I error = 7.9%, z = 4.21, p = 5.7×10^−5^) for scenarios i and ii, respectively. In the mixed scenario (iii), the type I error became significantly different to 5% for all N_F_/n ratio larger than 0.075 (type I error = 7.1%, z = 3.047, p = 0.0038). More details are available in File S1 and Fig. S2. Taken together, these results show that, as a rule of thumb, the logistic regression approach is robust for N_F_/n ratios below 0.1 for all types of factors.

### 2. Feline viruses associations

The two approaches (corrected chi-square and logistic regression) were used for the analysis of the interactions between four cat viruses (Table 2).

**Table 2:** Pearson's chi-square tests, corrected chi-square tests and logistic regressions for the search of viruses' interactions.

				No correction		Correction by known risk factors					
				Pearson's χ^2^		Corrected χ^2^				Logistic regressions	
Viruses	n^a^	N_F_ b	N_F_/n	χ^2^	P	χ^2^_corr_	ĉ^c^	P-value1	P-value2	Response	P
**FIV-FHV**	468	22	0.05	9.77	0.002	2.24	0.57	0.05	0.05	FIV	0.1
										FHV	0.05
**FIV-FCV**	465	26	0.06	12.72	<0.01	1.46	0.68	0.14	0.15	FIV	0.14
										FCV	0.11
**FIV-FPV**	469	23	0.05	1.36	0.244	0.68	0.48	0.23	0.23	FIV	0.28
										FPV	0.23
**FHV-FCV**	467	22	0.05	50.09	<0.01	20.81	0.66	1.9×10^−8^	0	FHV	1.2×10^−8^
										FCV	2.1×10^−8^
**FHV-FPV**	469	20	0.04	45.35	<0.01	54.26	0.65	0	0	FHV	<2.2×10^−16^
										FPV	<2.2×10^−16^
**FCV-FPV**	467	22	0.05	21.12	<0.01	26.39	0.58	1.7×10^−11^	0	FCV	4.0×10^−13^
										FPV	3.4×10^−12^
**FHV-FCV | FPV**	467	23	0.05			4.22	0.51	0.004	0.003	FHV	0.002
										FCV	0.03
**FHV-FPV | FCV**	467	21	0.04			35.94	0.58	2.7×10^−15^	0	FHV	<2.2×10^−16^
										FPV	<2.2×10^−16^
**FCV-FPV | FHV**	467	23	0.05			11.55	0.48	1×10^−06^	0	FCV	3.5×10^−7^
										FPV	1.1×10^−6^

a sample size;

b number of factors;

c dispersion coefficient.

At the bottom of the table, the significant interactions were tested for a possible confounding effect of the status to the third virus (e.g., FHV-FCV | FPV studies the association between FHV-FCV after correction by FPV).

Results showed that the interaction was not significant for pairs involving FIV. All other pairs (FHV-FCV, FHV-FPV and FCV-FPV) were found to interact, i.e., the number of individuals co-infected by two viruses could not be explained by shared risk factors. The three significant associations were all positive, meaning that there were always more co-infected individuals than expected considering shared risk factors (Table 3).

**Table 3:** Observed (O) and expected (E) frequencies under the independence hypothesis considering risk factors.

	−/−		+/−		−/+		+/+	
Viruses	O	E	O	E	O	E	O	E
FIV-FHV	160	158	16	21	232	237	57	52
FIV-FCV	78	75	2	5	314	317	71	68
FIV-FPV	302	299	51	54	94	97	22	19
FHV-FCV	59	40	22	41	119	138	**267**	248
FHV-FPV	165	131	187	220	14	48	**103**	69
FCV-FPV	77	58	273	292	4	23	**113**	94

The number of double seronegative (−/−), single seropositive (+/−, −/+) and double seropositive cats (+/+) are presented for the six tested pairs of feline viruses. More co-infected cats than expected were observed for the three significantly associated pairs (in bold).

Pairwise interactions between FHV, FCV and FPV could have come from the fact that one virus was a common risk factor for the two others. This possibility was tested (see the three last lines of Table 2) by adding the serological status to one virus as a common risk factor for the two others. Results led to reject this hypothesis, meaning that the observed associations cannot be solely explained by the fact that one virus interacts with the two others.

The two P-values obtained for the corrected chi-squares are coherent. As for the P-values obtained for the logistic regression approach, they are usually slightly lower than those of the corrected chi-squares, probably because of the over-predictive trend of logistic regressions.

In addition, as with simulated data, the logistic regression approach was less robust to small sample sizes than the corrected chi-square (Table S1). This was tested by randomly sampling smaller subsets of the cat data in order to increase the N_F_/n ratio.

Finally, to emphasise the need to consider risk factors in the analysis of interactions, we also calculated the classical independence Pearson's chi-square. This approach, which does not integrate risk factors, predicted an association between five of the six tested pairs. In the case of the FIV-FCV and FIV-FHV pairs, it would lead to wrongly conclude on the existence of an interaction, whereas the two approaches have shown that these apparent interactions were in fact explicable by shared factors.

## Discussion

Common risk factors can create statistical associations. This work confirmed that ignoring them would lead to wrong conclusions. Ignoring them would indeed result in an over-estimation of the number of interactions as any association, biological or statistical, would be put in one basket. The loss of significance after controlling for other factors was illustrated in this paper with feline viruses data, and was previously found by Behnke et al. [39] for helminth parasites of the wood mice. The next step was to identify an appropriate way to take those risk factors into account.

### 1. Logistic regression approach *versus* corrected chi-square tests

Two approaches to take risk factors into account with serological data (i.e., presence-absence) were proposed and examined. Those are the use of logistic regression models as previously done by some authors [52], [57], or an adaptation of the chi-square test for independence presented for the first time in this paper.

To determine which method should be used under which circumstances, we need to make the following considerations. First, the corrected chi-square involves 2n+2 estimations of the logistic regression coefficients, n being the number of bootstraps. In comparison, only two models must be parameterized in the logistic regression. As a consequence, the logistic regression approach is much faster to run (less than a second *versus* 2.5 minutes for the corrected chi-square for a model with 6 factors in full interaction and 300 individuals, for 1000 bootstraps, using a desktop computer with an Intel(R) core(TM)2 Quad CPU Q6600 processor). Second, the corrected chi-square is more robust than the logistic regression, especially for small sample size. A first solution would be to use the corrected chi-square as soon as simulation times are acceptable. For a 5% rejection threshold, a more straightforward alternative is to use the corrected chi-square by default as soon as the ratio between the sample size and the number of parameters is below 10 and the logistic regression in the opposite case. However, we did not test all potential situations and further analyses are needed to determine the limit of robustness of the logistic regression approach (in particular in situations where the probability of infection is not 50% and can be affected by risk factors).

Two P-values have been proposed for the corrected chi-square. The first one relies on the assumption that the corrected chi-square is proportional to a chi-square with one degree of freedom; the second one simply counts the proportion of *in silico* datasets for which the value of the corrected chi-square is above the observed value. Both P-values led to consistent results using a 5% rejection threshold, consistently with the fact that for all tested pairs the corrected chi-square fitted well with an under-dispersed chi-square with one degree of freedom (Fig. S1, Fig. S2). Which one should be used in practice actually depends on the simulation time. If simulations are fast enough and if running 1000 bootstrap is acceptable, P-value2 should be preferred. In the opposite case, a good option is to run much less bootstraps (typically 30) and to use P-value1.

Even if other alternative methods allow taking covariates into account, we only compared the corrected chi-square to the logistic regression approach. We could have compared it as well to log-linear models, which model the probability of infection with single and multiple parasite species from contingency tables and allow including known risk factors. However, in this approach the independence between parasites is tested using likelihood ratio tests, which are based on an asymptotic approximation of the deviance as in the logistic regression approach. They should therefore have the same limitations than logistic regressions and their robustness should be similarly influenced by the N_F_/n ratio. In addition, continuous variables are usually discretized in log-linear models, whereas the corrected chi-square allows working with continuous data.

### 2. Interactions between pairs of feline viruses

After correction by the known risk factors of the viruses, three pairs of feline viruses out of six appeared to be significantly associated. The N_F_/n ratio being 0.04 to 0.06, the logistic regression approach can be considered robust, at least for a 5% rejection threshold.

First, it is worth noting that age is a crucial covariate. The infection probability of all viruses increases with host' age [62], thus age must strongly participate in the generation of false interactions. This age-dependence is due to both a biological effect (i.e., behaviors and immune defenses may evolve with age, [82], [83]) and a mechanical effect (i.e., older individuals are more likely to be seropositive because of a longer exposure time). Disentangling both effects would require the use of Susceptible-Infected-Recovered (SIR) models, but was not necessary here. Indeed, to correct for age in the study of interactions, the important is to model the evolution of the probability of infection with age.

Correcting for all risk factors, no pair of viruses involving the Feline Immunodeficiency Virus (FIV-FHV, FIV-FCV, FIV-FPV) was significantly associated. This result is at first surprising because, as in humans infected by HIV, feline AIDS is characterised by a chronic immunodeficiency, allowing subsequent opportunistic infections (review in [84]). Indeed, although FIV positive cats can mount immune responses to administered antigens other than during the terminal phase of infection, their primary immune responses may be delayed or diminished [85], [86]. Experimental studies also revealed that cats co-infected by FIV and FCV or FHV had more severe disease signs than non-FIV infected cats [87], [88]. In addition, the presence of FHV was shown to accelerate FIV transcription through the activation of the FIV long terminal repeat [89], a phenomenon that was also shown *in vitro* for the human versions of the viruses, HSV2 and HIV [90]–[93]. Those laboratory experiments show that FIV infection may increase the severity of FHV or FCV-induced clinical signs but do not address the question of the effect of FIV on the sensitivity to FHV or FCV infection. Furthermore, the few epidemiological studies interested in the question did not demonstrate any epidemiological association between FIV and FHV [94]. In other words, if experimental investigations suggest a synergy between FIV and FHV and between FIV and FCV towards a more severe disease, our sero-epidemiological study suggests that the identified risk factors explain by themselves the apparent increase of double sero-positive individuals.

As for the FIV-FPV pair, this study is to our knowledge the first to search for a potential association. Whether risk factors were taken into account or not, we did not find any significant association between the two viruses. Again, this could be at first surprising as both viruses are supposed to be immunosuppressive [84], [95], [96]. In experimental conditions, FPV infection is more severe in FIV-infected cats [97]. Consequently, a positive association could have been expected if infections had facilitated each other (leading to numerous co-infections) or a negative association if the co-infection had led to a strong host mortality (leading to few co-infections). However, the FPV-induced decrease in the immune response is transient and more likely to occur in young kittens, whereas FIV infection is more frequent in adult cats. The persistence of FPV-antibodies can be longer than 7 years [98], and consequently, double seropositivity against FPV and FIV is not synonymous of co-infection. It is likely that co-infections by the two viruses are not frequent and mainly occur in adult animals which are less sensitive to FPV.

As no association was evidenced for these three pairs of viruses, the FIV infection does not seem to modify the risk of infection by another virus. However, our results do not exclude the occurrence of an interaction once both parasites are in contact within the host (e.g., directly through competition or indirectly via the host immune system), as suggested by several experimental co-infection studies. In addition, the FIV seropositivity status may encompass different stages of the infection with various degrees of immunodeficiency. The results of this study do not exclude the possibility that late stage FIV infection may increase the sensitivity to the other feline viruses.

On the contrary, the three other pairs (FHV-FCV, FHV-FPV and FCV-FPV) were significantly associated after correction by their known risk factors. It is to our knowledge the first evidence of a possible interaction between those viruses. As more double sero-positive cats than expected under the independence hypothesis were observed, possible synergies are suggested. After an acute infection, FHV is known to persist life-long in a latent form, which can be reactivated in stressful conditions [73]. Infection with FPV or FCV could thus be responsible for the reactivation of FHV in latently infected animals, resulting in seroconversion against both FHV and the new infecting virus. This could explain the FHV-FCV and FHV-FPV associations. In addition, since FPV is more immunosuppressive than FCV, the interaction between FPV and FHV is expected to be stronger than that between FCV and FHV, which is consistent with our results. The immunosuppressive effect of FPV could also explain the association with FCV. In that case however, contrary to FHV, it would require that the FCV-infection occurs at the time of the immunosuppression occuring within the two weeks post-FPV infection. Interestingly, a similar association between FPV and FCV antibodies was described in free-ranging lions in East Africa [99].

### 3. Real interactions or confounding factors?

This work pointed out new probable synergies between feline viruses that can now be further investigated in laboratory conditions. However, the associations could also result from the existence of an unknown confounding factor common to FHV, FCV and FPV. The feline parvovirus is immunosuppressive, as a result of the strong leukopenia occurring within the two weeks post-infection [95], [96]. This virus could therefore be a confounding factor to the FHV-FCV pair if FPV-seropositive cats are more susceptible to FHV and FCV at the same time. However, as shown in this paper, the FHV-FCV interaction remained significant after correction by FPV (Table 2).

If FPV is not a confounding factor, we cannot exclude the existence of another one, such as a greater susceptibility of certain individuals to infections whatever the parasite involved. Numerous studies have shown that an extensive inter-individual variability exists in response to certain pathogens, such as HIV (review in [100]), trypanosomiasis (review in [101]), or human and bovine tuberculosis (reviews in [102], [103]), including variations in susceptibility to the parasite, its transmission, and/or the course of disease progression. It has been attributed to host determinants and variability in multiple genes that regulate virus cell entry, acquired and innate immunity (e.g., macrophages, molecular and cellular actors of the inflammatory reaction), and others that influence the outcome of the infection. Hosts with a diminished or delayed innate immune response may in fact be more susceptible to any infection, with physiological parameters, such as hormonal profiles (e.g., [104]), possibly playing a role in the modulation of transmission efficiency and/or in the immune response intensity. A weaker physical condition could also lead to a higher sensitivity to infectious agents (lower dose-effect, different intra-host dynamic) (e.g., [105]). More generally, individuals' personality may as well be involved [61], [106]. A better understanding of genetic, physiological and immunological basis of such inter-individual variability would therefore be of particular interest in the context of polyparasitism. Another perspective of this work is the development of new methods able to distinguish pairwise interactions from those due to common confounding factors shared by the three viruses. Such methods could use the proportion of infected individuals that are in reality triply infected.

### 4. Conclusion

While the study of macroparasites usually uses quantitative data (i.e., parasite load per individual host), the study of microparasites on the field is most of the time limited to presence-absence data (i.e., serology), making the detection of associations between parasites more complicated from a methodological point of view. The corrected chi-square proposed in this study is, with the logistic regression approach, currently one of the rare ways to search for interaction between parasites from presence-absence data. This work provides evidence of the efficiency of such methods to reduce the bias introduced by common risk factors and encourages their use. However it also points out the low robustness of the likelihood ratio test for certain data characteristics. The corrected chi-square test must indeed be preferred for small sample size.

Those methods can be applied to any epidemiological study based on serology, within human or animal host populations. Applied here to feline viruses, they revealed significant associations between three pairs of feline viruses. If they still do not allow us to decide whether such associations are really true interactions or whether they reveal the existence of “over-susceptible” hosts, we believe it is an important step forward as it offers the possibility to point out parasites associations that should be further investigated in experimental conditions. The understanding of parasites interactions and of their consequences on diseases evolution, emergence and management is indeed a crucial challenge for human and animal epidemiologists of our time.

## Supporting Information

Figure S1 **Cumulative distribution of the corrected chi-square.** Cumulative distribution of the corrected chi-square obtained by parametric bootstrap considering the known risk factors for each pair of viruses (A: FIV-FHV; B: FIV-FCV; C: FIV-FPV; D: FHV-FCV; E: FHV-FPV; F: FCV-FPV) and considering the known risk factors and the serological status of a third virus (G: FHV-FCV | FPV; H: FHV-FPV | FCV; I: FCV-FPV | FHV). Thick blue line: empirical cumulative function of the corrected chi-square; thin black line: cumulative distribution function for a chi-square with one degree of freedom; dashed red line: empirical cumulative function of the corrected chi-square divided by the dispersion coefficient (ĉ). The fact that the thin solid and dashed lines are almost confounded shows that the corrected chi-square is proportional to a chi-square with one degree of freedom.
(EPS)

Figure S2 **Issue of the conformity tests of the type I error to 5% according to the N_F_/n ratio for the logistic regression approach.** The issue was coded 1 when the test was significant, 0 when not and the resulting logistic regression was drawn (dark line). Three scenarios are considered: i) all factors are qualitative (A); ii) all factors are quantitative (B) and iii) half of the factors are quantitative and the other half are qualitative (mixed scenario, C).
(EPS)

Figure S3 **Type I error (%) of the corrected chi-square tests according to the N_F_/n ratio and the type of P-value used for the corrected chi-square: P-value1 (blue empty points) or P-value2 (red full points).** Three scenarios are considered: i) all factors are qualitative (A); ii) all factors are quantitative (B) and iii) a half of the factors is quantitative and the other half is qualitative (mixed scenario, C). The dashed horizontal line represents a type I error of 5%.
(EPS)

Table S1 **Corrected chi-square tests and logistic regressions to search for feline viruses' interactions using subsets randomly sampled in cat data such that the N_F_/n ratio takes various values.**
(DOC)

File S1 **Robustness of the logistic regression approach and of the corrected chi-square test.** (1) Conformity tests of the type I error to 5%, (2) Influence of the way to calculate the P-value of the corrected chi-square test on the robustness of the study.
(DOC)

File S2 **“Chi2corr”, an R program for the application of the corrected chi-square test to any presence-absence data: test statistic, observed and expected frequencies, estimated dispersion coefficient (parametric bootstrap), P-values and distribution of the bootstrapped corrected chi-square.**
(R) Click here for additional data file.

File S3 **A step-by-step example of application of the corrected chi-square test to search for interaction between two parasites, using a provided dataset (“data_example.txt”, File S4) and the provided R program (“Chi2corr.R”, File S2).**
(DOC)

File S4 **“Data_example”, a generated dataset provided to test the R program.** It is made of 4 risk factors (2 quantitative and 2 qualitative factors), 2 serological statuses and 100 individuals.
(TXT)

## References

[1] Cox FEG (2001). Concomitant infections, parasites and immune responses.. Parasitology.

[2] Grmek MD (1969). Préliminaires d'une étude historique des maladies. Annales.. Economies, Sociétés, Civilisations.

[3] Rodriguez M, Terrazas LI, Marquez R, Bojalil R (1999). Susceptibility to *Trypanosoma cruzi* is modified by a previous non-related infection. Parasite Immunol..

[4] Lello J, Boag B, Fenton A, Stevenson IR, Hudson PJ (2004). Competition and mutualism among the gut helminths of a mammalian host.. Nature.

[5] Graham AL, Lamb TJ, Read AF, Allen JE (2005). Malaria-filaria coinfection in mice makes malarial disease more severe unless filarial infection achieves patency.. J Infect Dis.

[6] Abu-Raddad LJ, Patnaik P, Kublin JG (2006). Dual infection with HIV and malaria fuels the spread of both diseases in sub-Saharan Africa.. Science.

[7] Graham AL (2008). Ecological rules governing helminth-microparasite coinfection.. Proc Natl Acad Sci U S A.

[8] Graham AL (2002). When T-helper cells don't help: immunopathology during concomitant infections.. The Quarterly Review of Biology.

[9] Graham AL, Cattadori IM, Lloyd-Smith JO, Ferrari MJ, Bjornstad ON (2007). Transmission consequences of coinfection: cytokines writ large?. Trends Parasitol.

[10] Christensen NO, Nansen P, Fagbemi BO, Monrad J (1987). Heterologous antagonistic and synergistic interactions between helminths and between helminths and protozoans in concurrent experimental infection of mammalian hosts.. Parasitol Res.

[11] Behnke JM, Bajer A, Sinski E, Wakelin D (2001). Interactions involving intestinal nematodes of rodents: experimental and field studies.. Parasitology.

[12] Pedersen AB, Fenton A (2006). Emphasizing the ecology in parasite community ecology.. Trends Ecol Evol.

[13] Bentwich Z, Kalinkovich A, Weisman Z, Borkow G, Beyers N (1999). Can eradication of helminthic infections change the face of AIDS and tuberculosis?. Immunology Today.

[14] Hoffman IF, Jere CS, Taylor TE, Munthali P, Dyer JR (1999). The effect of Plasmodium falciparum malaria on HIV-1 RNA blood plasma concentration.. Aids.

[15] Whitworth J, Morgen D, Quigley M, Smith A, Mayanja S (2000). Effect of HIV-1 and increasing immunosuppression on malaria parasitaemia and clinical episodes in adults in rural Uganda: a cohort study.. Lancet.

[16] Corbett EL, Steketee RW, ter Kuile FO, Latif AS, Kamali A (2002). HIV-1/AIDS and the control of other infectious diseases in Africa.. Lancet.

[17] Corbett EL, Watt CJ, Walker N, Maher D, Williams BG (2003). The growing burden of tuberculosis - Global trends and interactions with the HIV epidemic.. Arch Intern Med.

[18] Celum CL (2004). The interaction between herpes simplex virus and human immunodeficiency virus.. Herpes.

[19] Weiss E (2004). Epidemiology of herpes simplex virus type 2 infection in the developing world.. Herpes.

[20] Maher D, Harries A, Getahun H (2005). Tuberculosis and HIV interaction in sub-Saharan Africa: impact on patients and programmes; implications for policies.. Trop Med Int Health.

[21] Nacher M (2002). Worms and malaria: noisy nuisances and silent benefits.. Parasite immunol.

[22] Sokhna C, Le Hesran JY, Mbaye PA, Akiana J, Camara P (2004). Increase of malaria attacks among children presenting concomitant infection by *Schistosoma mansoni* in Senegal.. Malaria J.

[23] Druilhe P, Tall A, Sokhna C (2005). Worms can worsen malaria: towards a new means to roll back malaria?. Trends Parasitol.

[24] Mwangi TW, Bethony JM, Brooker S (2006). Malaria and helminth interactions in humans: an epidemiological viewpoint.. Ann Trop Med Parasit.

[25] Lotz JM, Font WF (1991). The role of positive and negative interspecific associations in the organization of communities of intestinal helminths of bats.. Parasitology.

[26] Stancampiano L, Gras LM, Poglayen G (2010). Spatial niche competition among helminth parasites in horse's large intestine.. Vet Parasitol.

[27] Bush AO, Holmes JC (1986). Intestinal Helminths Of Lesser Scaup Ducks - Patterns Of Association.. Can J Zool.

[28] Karvonen A, Seppälä O, Valtonen ET (2009). Host immunization shapes interspecific associations in trematode parasites.. J Anim Ecol.

[29] Schall JJ, Bromwich CR (1994). Interspecific interactions tested - 2 species of malarial parasite in a west-african lizard.. Oecologia.

[30] McNab T, Desport M, Dobson R, Tenaya MIW, Hartaningsih N (2010). Prior bovine immunodeficiency virus infection does not inhibit subsequent superinfection by the acutely pathogenic Jembrana disease virus.. Virology.

[31] Harris JB, Podolsky MJ, Bhuiyan TR, Chowdhury F, Khan AI (2009). Immunologic responses to *Vibrio cholerae* in patients co-infected with intestinal parasites in Bangladesh.. PLoS Negl Trop D.

[32] Pontier D, Fouchet D, Bahi-Jaber N, Poulet H, Guiserix M (2009). When domestic cat (*Felis silvestris catus*) population structures interact with their viruses.. C R Biol.

[33] Behnke JM, Wakelin D, Wilson MM (1978). *Trichinella spiralis*: delayed rejection in mice concurrently infected with *Nematospiroides dubius*.. Exp Parasitol.

[34] Adams DB, Anderson BH, Windon RG (1989). Crossimmunity between *Haemonchus contortus* and *Trichostrongylus colubriformis* in sheep.. Int J Parasitol.

[35] Frontera E, Alcaide A, Dominguez-Alpizar JL, Boes J, Reina D (2005). Evidence of interaction between *Ascaris suum* and *Metastrongylus apri* in experimentally infected pigs.. Vet Parasitol.

[36] Kuris AM, Lafferty KD (1994). Community structure: larval trematodes in snail hosts.. Annual Rev Ecol Syst.

[37] Lafferty KD, Sammond DT, Kuris AM (1994). Analysis of larval trematode communities.. Ecology.

[38] Haukisalmi V, Henttonen H (1998). Analysing interspecific associations in parasites: alternative methods and effects of sampling heterogeneity.. Oecologia.

[39] Behnke JM, Gilbert FS, Abu-Madi MA, Lewis JW (2005). Do the helminth parasites of wood mice interact?. J Anim Ecol.

[40] Krasnov BR, Stanko M, Morand S (2006). Are ectoparasite communities structured? Species co-occurrence, temporal variation and null models.. J Anim Ecol.

[41] Hoste H, Cabaret J (1992). lntergeneric relations between nematodes of the digestive tract of lambs: a multivariate approach.. Int J Parasitol.

[42] Hoste H, Beveridge I (1993). Interspecific and intergeneric relations between nematodes parasitic in the stomachs of kangaroos and wallabies.. Trans R Sot South Aust.

[43] Bucknell D, Hoste H, Gasser RB, Beveridge I (1996). The structure of the community of strongyloid nematodes of domestic equids.. J Helminthol.

[44] Wasserheit JN (1992). Epidemiologic Synergy - Interrelationships between Human-Immunodeficiency-Virus infection and other sexually-transmitted diseases - (Reprinted From Aids And Womens Reproductive Health, Ch 5, 1992).. Sex Transm Dis.

[45] Worrall E, Basu S, Hanson K (2003). The relationship between socio-economic status and malaria: a review of the literature..

[46] Huang Y, Rohani P (2006). Age-structured effects and disease interference in childhood infections.. P Roy Soc B-Biol Sci.

[47] Haukisalmi V, Henttonen H (1993). Coexistence in helminthes of the bank vole *Clethrionomys glareolus*.1. Patterns of co-ccurrence.. J Anim Ecol.

[48] Poulin R (2001). Interactions between species and the structure of helminth communities.. Parasitology.

[49] Fenton A, Viney ME, Lello J (2010). Detecting interspecific macroparasite interactions from ecological data: patterns and process.. Ecol Lett.

[50] Telfer S, Lambin X, Birtles R, Beldomenico P, Burthe S (2010). Species Interactions in a Parasite Community Drive Infection Risk in a Wildlife Population.. Science.

[51] Bilge-Dagalp S, Can-Sahna K, Yildirm Y, Karaoglu T, Alkan F (2008). Effects of bovine leucosis virus (BLV) infection on the bovine viral diarrhoea virus (BVDV) and bovine herpes virus 1 (BHV1) seroprevalences in dairy herds in Turkey.. Rev Med Vet.

[52] Jolles AE, Ezenwa VO, Etienne RS, Turner WC, Olff H (2008). Interactions between macroparasites and microparasites drive infection patterns in free-ranging African buffalo.. Ecology.

[53] Lafferty K, Sammond D, Kuris A (1994). Analysis of larval trematode communities.. Ecology.

[54] Howard S, Donnelly C, Chan M (2001). Methods for estimation of associations between multiple species parasite infections.. Parasitology.

[55] Behnke JM, Gilbert FS, Abu-Madi MA, Lewis JW (2005). Do the helminth parasites of wood mice interact?. Journal Of Animal Ecology.

[56] Faulkner H, Turner J, Behnke J, Kamgno J, Rowlinson M (2005). Associations between filarial and gastrointestinal nematodes.. Transactions of the Royal Society of Tropical Medicine and Hygiene.

[57] Marchandeau S, Letty J, Bertagnoli S, Peralta B, Boucraut-Baralon C (2004). Possible interaction between myxomatosis and calicivirosis related to rabbit haemorrhagic disease affecting the European rabbit.. Vet record.

[58] Liberg O (1980). Spacing pattern in a population of rural free roaming domestic cats.. Oikos.

[59] Say L, Devillard S, Natoli E, Pontier D (2002). The mating system of feral cats (*Felis catus L.*) in a sub-Antarctic environment.. Polar Biol.

[60] Pontier D, Fromont E, Courchamp F, Artois M, Yoccoz NG (1998). Retroviruses and sexual size dimorphism in domestic cats (*Felis catus L.*).. P Roy Soc B-Biol Sci.

[61] Natoli E, Say L, Cafazzo S, Bonanni R, Schmid M (2005). Bold attitude makes male urban feral domestic cats more vulnerable to Feline Immunodeficiency Virus.. Neurosc Biobehav R.

[62] Hellard E, Fouchet D, Santin-Janin H, Tarin B, Badol V (2011). When cats' ways of life interact with their viruses: a study in 15 natural populations of owned and unowned cats (*Felis silvestris catus*).. Prev Vet Med.

[63] Wilks SS (1938). The Large-Sample Distribution of the Likelihood Ratio for Testing Composite Hypotheses.. The Annals of Mathematical Statistics.

[64] Courchamp F, Pontier D (1994). Feline immunodeficiency virus: an epidemiologic review.. C R Acad Sci III, Sci Vie.

[65] Ostrowski S, Van Vuuren M, Lenain DM, Durand A (2003). A Serologic survey of wild felids from Central West Saudi Arabia.. J Wildl Dis.

[66] Troyer JL, Pecon-Slattery J, Roelke ME, Johnson W, VandeWoude S (2005). Seroprevalence and genomic divergence of circulating strains of Feline Immunodeficiency Virus among *Felidae* and *Hyaenidae* species.. J Virol.

[67] Sparger EE (1993). Current thoughts on feline immunodeficiency virus infection.. Vet Clin North Am Small Anim Pract.

[68] Bendinelli M, Pistello M, Lombardi S, Poli A, Garzelli C (1995). Feline immunodeficiency virus: an interesting model for AIDS studies and an important cat pathogen.. Clin Microbiol Rev.

[69] Radford AD, Addie D, Belák S, Boucraut-Baralon C, Egberink H (2009). Feline calicivirus infection: ABCD guidelines on prevention and management.. J Feline Med Surg.

[70] Thiry E, Addie D, Belák S, Boucraut-Baralon C, Egberink H (2009). Feline herpesvirus infection: ABCD guidelines on prevention and management.. J Feline Med Surg.

[71] Povey RC, Johnson RH (1970). Observations on the epidemiology and control of viral respiratory disease in cats.. J Small Anim Pract.

[72] Gaskell RM, Povey RC (1982). Transmission of feline viral rhinotracheitis.. Vet Rec.

[73] Gaskell RM, Povey RC (1977). Experimental induction of feline rhinotracheitis virus re-excretion in FVR-recovered cats.. Vet Rec.

[74] Steinel A, Parrish CR, Bloom ME, Truyen U (2001). Parvovirus infections in wild carnivores.. J Wildl Dis.

[75] Truyen U, Addie D, Belak S, Boucraut-Baralon C, Egberink H (2009). Feline panleukopenia ABCD guidelines on prevention and management.. J Feline Med Surg.

[76] Csiza CK, Scott FW, De Lahunta A, Gillepsie JH (1971). Immune carrier state of feline panleukopenia virus-infected cats.. Am J Vet Res.

[77] Reif JS (1976). Seasonality, mortality and herd immunity in feline panleukopenia.. Am J Epidemiol.

[78] Berthier K, Langlais M, Auger P, Pontier D (2000). Dynamics of a feline virus with two transmission modes within exponentially growing host populations.. P Roy Soc B-Biol Sci.

[79] Fouchet D, Leblanc G, Sauvage F, Guiserix M, Poulet H (2009). Using Dynamic Stochastic Modelling to Estimate Population Risk Factors in Infectious Disease: The Example of FIV in 15 Cat Populations.. Plos One.

[80] Poulet H (2007). Alternative early life vaccination programs for companion animals.. J Comp Pathol.

[81] Anderson DR, Burnham KP, White GC (1994). AIC model selection in overdispersed capture-recapture data.. Ecology.

[82] Levy O (2007). Innate immunity of the newborn: basic mechanisms and clinical correlates.. Nat Rev Immunol.

[83] Nakamichi M (2003). Age-related differences in social grooming among adult female Japanese monkeys (*Macaca fuscata*).. Primates.

[84] Willett BJ, Flynn JN, Hosie MJ (1997). FIV infection of the domestic cat: An animal model for AIDS.. Immunology Today.

[85] Dawson S, Smyth NR, Bennett M, Gaskell RM, McCracken CM (1991). Effect of primary stage feline immunodeficiency virus infection on subsequent feline calicivirus vaccination and challenge in cats.. AIDS.

[86] Reubel GH, Dean GA, George JW, Barlough JE, Pedersen NC (1994). Effects of incidental infections and immune activation on disease progression in experimentally feline immunodeficiency virus-infected cats.. J Acquir Immun Defic Syndr.

[87] Tenorio AP, Franti CE, Madewell BR, Pedersen NC (1991). Chronic oral infections of cats and their relationship to persistent oral carriage of feline calici-, immunodeficiency, or leukemia viruses.. Vet Immunol Immunop.

[88] Reubel GH, George JW, Barlough JE, Higgins J, Grant CK (1992). Interaction of acute feline herpesvirus-1 and chronic feline immunodeficiency virus infections in experimentally infected specific pathogen free cats.. Vet Immunol Immunop.

[89] Kawaguchi Y, Miyazawa T, Horimoto T, Itagaki SI, Fukasawa M (1991). Activation of feline immunodeficiency virus long terminal repeat by feline herpesvirus type 1.. Virology.

[90] Mosca JD, Bednarik DP, Raj NBK, Rosen CA, Sodroski JG (1987). Herpes simplex virus type-1 can reactivate transcription of latent human immunodeficiency virus.. Nature.

[91] Albrecht MA, Deluca NA, Byrn RA, Schaffer PA, Hammer SM (1989). The Herpes-simplex Virus Immediate-early Protein, Icp4, Is Required To Potentiate Replication of Human Immunodeficiency Virus In Cd4+ Lymphocytes.. J Virol.

[92] Golden MP, Kim SY, Hammer SM, Ladd EA, Schaffer PA (1992). Activation of Human-immunodeficiency-virus By Herpes-simplex Virus.. J Infect Dis.

[93] Heng MCY, Heng SY, Allen SG (1994). Co-infection and synergy of human immunodeficiency virus-1 and herpes simplex virus-1.. Lancet.

[94] Knowles JO, Gaskell RM, Gaskell CJ, Harvey CE, Lutz H (1989). Prevalence Of Feline Calicivirus, Feline Leukemia-Virus And Antibodies To FIV In Cats With Chronic Stomatitis.. Vet Record.

[95] Pedersen NC, Appel MJ (1987). Feline panleukopenia virus.. Virus infections of carnivores.

[96] Ikeda Y, Shinozuka J, Miyazawa T, Kurosawa K, Izumiya Y (1998). Apoptosis in feline panleukopenia virus-infected lymphocytes.. J Virol.

[97] Buonavoglia C, Marsilio F, Tempesta M, Buonavoglia D, Tiscar PG (1993). Use of a feline panleukopenia modified live virus vaccine in cats in the primary-stage of feline immunodeficiency virus infection.. Zentralbl Veterinarmed B.

[98] Scott FW, Geissinger CM (1999). Long-term immunity in cats vaccinated with an inactivated trivalent vaccine.. Am J Vet Res.

[99] Hofmann-Lehmann R, Fehr D, Grob M, Elgizoli M, Packer C (1996). Prevalence of antibodies to feline parvovirus, calicivirus, herpesvirus, coronavirus, and immunodeficiency virus and of feline leukemia virus antigen and the interrelationship of these viral infections in free-ranging lions in east Africa.. Clin Diagn Lab Immunol.

[100] Kaur G, Mehra N (2009). Genetic determinants of HIV-1 infection and progression to AIDS: immune response genes.. Tissue Antigens.

[101] Courtin D, Berthier D, Thevenon S, Dayo GK, Garcia A (2008). Host genetics in African trypanosomiasis.. Infect Genet Evol.

[102] Kramnik I (2008). Genetic dissection of host resistance to *Mycobacterium tuberculosis*: the sst1 locus and the Ipr1 gene.. Immunology, Phenotype First: How Mutations Have Established New Principles and Pathways In Immunology.

[103] Allen AR, Minozzi G, Glass EJ, Skuce RA, McDowell SWJ (2010). Bovine tuberculosis: the genetic basis of host susceptibility.. P Roy Soc B-Biol Sci.

[104] Folstad I, Karter AJ (1992). Parasites, bright males, and the immunocompetence handicap.. Am Nat.

[105] Beldomenico PM, Telfer S, Lukomski L, Gebert S, Bennett M (2009). Host condition and individual risk of cowpox virus infection in natural animal populations: cause or effect?. Epidemiol Infect.

[106] Reale D, Garant D, Humphries MM, Bergeron P, Careau V (2010). Personality and the emergence of the pace-of-life syndrome concept at the population level.. Philos T R Soc B.

