# Supplementary material for: True versus False Parasite Interactions: A Robust Method to Take Risk Factors into Account and Its Application to Feline Viruses
Source: PLoS One. 2012 Jan 3;7(1):e29618. doi: 10.1371/journal.pone.0029618 (PMC3250451; doi:10.1371/journal.pone.0029618)
Supplement: Table S1 — Corrected chi-square tests and logistic regressions to search for feline viruses' interactions using subsets randomly sampled in cat data such that the NF/n ratio takes various values. (DOC) [file pone.0029618.s004.doc]

**Table S1. Corrected chi-square tests and logistic regressions to search for feline viruses’ interactions using subsets randomly sampled in cat data such that the N_F_/n ratio takes various values.**

|  |  |  |  | **chi2corr** | | | | | | **Logistic regressions** | | |
| --- | --- | --- | --- | --- | --- | --- | --- | --- | --- | --- | --- | --- |
| **viruses** | **n^a^** | **N_F_^b^** | **N_F_/n** | **χ²corr** | **ĉ^c^** | **P-value1** | **F_1_^d^** | **P-value2** | **F_2_^d^** | **Response** | **P** | **F_3_^d^** |
|  |  |  |  |  |  |  |  |  |  |  |  |  |
| **FIV/FHV** | 400 | 22 | 0.06 | 2.22 | 0.54 | 0.06 | 0.05 | 0.041 | 0.11 | FIV | 0.08 | 0.22 |
|  |  |  |  |  |  |  |  |  |  | FHV | 0.04 | 0.21 |
|  | 300 | 22 | 0.07 | 0.9 | 0.52 | 0.18 | 0.27 | 0.194 | 0.35 | FIV | 0.78 | 0.24 |
|  |  |  |  |  |  |  |  |  |  | FHV | 0.78 | 0.29 |
|  | 200 | 22 | 0.11 | 1.45 | 0.49 | 0.10 | 0.16 | 0.086 | 0.21 | FIV | 0.08 | 0.21 |
|  |  |  |  |  |  |  |  |  |  | FHV | 0.08 | 0.17 |
|  | 100 | 22 | 0.22 | 1.0 | 0.49 | 0.15 | 0.08 | 0.1 | 0.13 | FIV | 0.58 | 0.3 |
|  |  |  |  |  |  |  |  |  |  | FHV | 0.11 | 0.25 |
|  |  |  |  |  |  |  |  |  |  |  |  |  |
| **FIV/FCV** | 400 | 26 | 0.07 | 2.16 | 0.64 | 0.08 | 0.04 | 0.07 | 0.04 | FIV | 0.04 | 0.06 |
|  |  |  |  |  |  |  |  |  |  | FCV | 0.03 | 0.13 |
|  | 300 | 26 | 0.09 | 1.64 | 0.69 | 0.13 | 0.03 | 0.11 | 0.07 | FIV | 4.5x10^-3^ | 0.13 |
|  |  |  |  |  |  |  |  |  |  | FCV | 1.8x10^-3^ | 0.13 |
|  | 200 | 26 | 0.13 | 3.01 | 0.56 | 0.04 | 0.05 | 0.01 | 0.11 | FIV | 0.49 | 0.22 |
|  |  |  |  |  |  |  |  |  |  | FCV | 0.30 | 0.21 |
|  | 100 | 26 | 0.26 | 0.07 | 1.3x10^-11^ | 1 | 0.03 | 0.26 | 0.1 | FIV | 0.14 | 0.28 |
|  |  |  |  |  |  |  |  |  |  | FCV | 0.99 | 0.24 |
|  |  |  |  |  |  |  |  |  |  |  |  |  |
| **FIV/FPV** | 400 | 23 | 0.06 | 0.05 | 0.45 | 0.52 | 0.01 | 0.74 | 0.07 | FIV | 0.28 | 0.03 |
|  |  |  |  |  |  |  |  |  |  | FPV | 0.30 | 0.01 |
|  | 300 | 23 | 0.08 | 0.06 | 0.45 | 0.50 | 0.02 | 0.72 | 0.04 | FIV | 0.23 | 0.08 |
|  |  |  |  |  |  |  |  |  |  | FPV | 0.07 | 0.08 |
|  | 200 | 23 | 0.12 | 1.81 | 0.39 | 0.06 | 0.02 | 0.03 | 0.05 | FIV | 0.25 | 0.03 |
|  |  |  |  |  |  |  |  |  |  | FPV | 0.26 | 0.04 |
|  | 100 | 23 | 0.23 | 1.31 | 0.17 | 0.04 | 0.09 | 0.006 | 0.12 | FIV | 0.32 | 0.18 |
|  |  |  |  |  |  |  |  |  |  | FPV | 0.18 | 0.11 |
|  |  |  |  |  |  |  |  |  |  |  |  |  |
| **FHV/FCV** | 400 | 22 | 0.06 | 13.57 | 0.61 | 9.4x10^-5^ | 1 | 0 | 1 | FHV | 1.6x10^-6^ | 1 |
|  |  |  |  |  |  |  |  |  |  | FCV | 1.8x10^-6^ | 1 |
|  | 300 | 22 | 0.07 | 11.37 | 0.55 | 2.4x10^-4^ | 1 | 0 | 1 | FHV | 6.4x10^-9^ | 1 |
|  |  |  |  |  |  |  |  |  |  | FCV | 1.3x10^-8^ | 1 |
|  | 200 | 22 | 0.11 | 9.21 | 0.55 | 8.7x10^-4^ | 0.97 | 0 | 0.97 | FHV | 2.1x10^-7^ | 1 |
|  |  |  |  |  |  |  |  |  |  | FCV | 5.9x10^-8^ | 0.98 |
|  | 100 | 22 | 0.22 | 6.60 | 0.39 | 2.5x10^-3^ | 0.67 | 0.001 | 0.74 | FHV | 0.02 | 0.76 |
|  |  |  |  |  |  |  |  |  |  | FCV | 0.32 | 0.68 |
|  |  |  |  |  |  |  |  |  |  |  |  |  |
| **FHV/FPV** | 400 | 20 | 0.05 | 51.90 | 0.62 | 1.9x10^-13^ | 1 | 0 | 1 | FHV | < 2.2x10^-16^ | 1 |
|  |  |  |  |  |  |  |  |  |  | FPV | < 2.2x10^-16^ | 1 |
|  | 300 | 20 | 0.07 | 39.91 | 0.69 | 1.1x10^-10^ | 1 | 0 | 1 | FHV | 1.1x10^-14^ | 1 |
|  |  |  |  |  |  |  |  |  |  | FPV | 3.4x10^-15^ | 1 |
|  | 200 | 20 | 0.1 | 26.36 | 0.57 | 9x10^-9^ | 1 | 0 | 1 | FHV | 1.2x10^-9^ | 1 |
|  |  |  |  |  |  |  |  |  |  | FPV | 1.9x10^-9^ | 1 |
|  | 100 | 20 | 0.2 | 7.57 | 0.35 | 1.29x10^-3^ | 0.99 | 0 | 1 | FHV | 1.3x10^-10^ | 0.99 |
|  |  |  |  |  |  |  |  |  |  | FPV | 1.2x10^-11^ | 1 |
|  |  |  |  |  |  |  |  |  |  |  |  |  |
| **FCV/FPV** | 400 | 22 | 0.06 | 23.52 | 0.58 | 4.1x10^-7^ | 1 | 0 | 1 | FCV | 9.9x10^-11^ | 1 |
|  |  |  |  |  |  |  |  |  |  | FPV | 2.3x10^-9^ | 1 |
|  | 300 | 22 | 0.07 | 12.43 | 0.59 | 1.6x10^-4^ | 1 | 0 | 1 | FCV | 1.7x10^-9^ | 1 |
|  |  |  |  |  |  |  |  |  |  | FPV | 8.6x10^-10^ | 1 |
|  | 200 | 22 | 0.11 | 13.55 | 0.58 | 8.6x10^-5^ | 1 | 0 | 1 | FCV | 1.6x10^-4^ | 1 |
|  |  |  |  |  |  |  |  |  |  | FPV | 1.2x10^-3^ | 1 |
|  | 100 | 22 | 0.22 | 5.70 | 0.31 | 3.2x10^-3^ | 0.8 | 0 | 0.92 | FCV | 6.2x10^-7^ | 0.97 |
|  |  |  |  |  |  |  |  |  |  | FPV | 3.7x10^-5^ | 0.94 |

^a^ sample size ; ^b^ number of factors ; ^c^ dispersion coefficient ; ^d^ proportion of P-values<0.05.

The values presented here come from a single resampling in cat data. P-value1 and P-value2 correspond to the two ways of calculating the P-value of the corrected chi-square test as described in the text. For each subset, a random resampling was applied 100 times and the proportion of significant tests (F_i_) was then estimated.
