# Supplementary material for: True versus False Parasite Interactions: A Robust Method to Take Risk Factors into Account and Its Application to Feline Viruses
Source: PLoS One. 2012 Jan 3;7(1):e29618. doi: 10.1371/journal.pone.0029618 (PMC3250451; doi:10.1371/journal.pone.0029618)
Supplement: File S1 — Robustness of the logistic regression approach and of the corrected chi-square test. (1) Conformity tests of the type I error to 5%, (2) Influence of the way to calculate the P-value of the corrected chi-square test on the robustness of the study. (DOC) [file pone.0029618.s005.doc]

**File S1**

**Robustness of the logistic regression approach and of the corrected chi-square test for the detection of interspecific parasite interaction in the field.**

1. **Conformity tests of the type I error to 5%**

The type I error (risk of wrongly deciding H1) of the logistic regression approach and of the corrected chi-square test is estimated using generated data and parametric bootstrap as described in the paper. The observed error is then compared to 5% by a conformity test.

The hypotheses of this test are:

H_0_: F=p *versus* H_1_: F≠p.

The statistic of this test is:

$$z=\frac{F-p}{\sqrt{\frac{pq}{n}}}$$

Where *F* is the observed proportion (type I error), *p* the theoretical proportion (0.05), *q*=1-p and *n* the sample size (number of bootstrap simulations). When the null hypothesis is true, z follows a standard normal distribution.

For each method, the conformity test was run for each value of the N_F_/n ratio (N_F_: number of model parameters; n: sample size), and for each scenario (i: only qualitative factors are included in the model, ii: only quantitative factors are included in the model, iii: quantitative and qualitative factors are included in the model).

The type I error of the corrected chi-square was not significantly different from 5%, except for very rare cases.

That of the logistic regression approach, on the contrary, rapidly became larger than 5% for increasing N_F_/n ratios, whatever the scenario. Therefore, to better see the influence of the N_F_/n ratio on the robustness of the regression approach, the issue of the tests was coded 1 when significant and 0 when non significant and graphically represented (Fig. S2). The predicted values of a logistic regression expressing the issue of the test in function of the N_F_/n ratio was added on the plot.

From figure S2 and the results of the tests, we can see that the type I error of the logistic regression approach becomes systematically significantly different from 5% for ratios larger than 0.12, 0.08 and 0.075 for scenarios i, ii and iii, respectively.

1. **Influence of the way to calculate the P-value of the corrected chi-square test on the robustness of the study**

As described in the paper, the value of the corrected chi-square was estimated by parametric bootstrap. Two ways of calculating the P-value were derived from this procedure. P-value1 was estimated assuming that the corrected chi-square is proportional to a one degree of freedom chi-square, the coefficient of over- (or under-) dispersion (ĉ) being defined by the mean of the bootstrapped corrected chi-square. P-value2 was given by the proportion of bootstrapped corrected chi-squares which were smaller than the observed value.

The robustness of the corrected chi-square test was studied considering both P-values, using generated data and parametric bootstrap.

Figure S3 shows that, using a 5% rejection threshold, both P-values are fine, i.e., they have a 5% probability of being below 0.05 when H0 is true.
