# Supplementary material for: True versus False Parasite Interactions: A Robust Method to Take Risk Factors into Account and Its Application to Feline Viruses
Source: PLoS One. 2012 Jan 3;7(1):e29618. doi: 10.1371/journal.pone.0029618 (PMC3250451; doi:10.1371/journal.pone.0029618)
Supplement: File S3 — A step-by-step example of application of the corrected chi-square test to search for interaction between two parasites, using a provided dataset (“data_example.txt”, File S4) and the provided R program (“Chi2corr.R”, File S2). (DOC) [file pone.0029618.s007.doc]

**File S3**

**A step-by-step example of application of the corrected chi-square to search for interaction between two parasites**, using a provided dataset (“data_example.txt ”, File S4) and the provided R program (“Chi2corr.R”, File S2).

1. **Data formatting, general information**

**1.1. Dataset and file format**

The used dataset must be a data frame including data for individuals tested for both studied parasites, with the observed values of the risk factors and serological status in columns and the individuals in lines. The extension of the data file must be “.txt”.

**1.2. Missing data management**

Any missing data must be indicated by “NA” in the corresponding case. Note that any other character or symbol or any number (e.g. “0”) will be considered as an existing value/modality by the software. Reciprocally, no factor modality must be named “NA” as it would be considered as a missing value.

**1.3. Required R packages**

Two R packages are required to run the *Chi2corr* function: *MASS* and *tcltk*. If you need to install them, please do it first (they are available on any CRAN mirror).

1. **Step-by-step example**

**2.1. The “data_example” dataset**

In the proposed example, the corrected chi-square test is applied to the “data_example” dataset, provided as File S4. The dataset is composed of four randomly generated risk factors, two quantitative (“F2”, “F4”, sampled in a standard normal distribution) and two qualitative (“F1”, “F3”, with three and two modalities, respectively), and of the serological status to two parasites (“parasite1”, “parasite2”, all individuals having an independent 0.5 probability of being seropositive for each pathogen). Missing data (“NAs”) have been added voluntarily.

**2.2. Application of the corrected chi-square test to the “data_example” dataset**

The R program “Chi2corr.R”, provided as File S2, is used to run 1000 bootstraps, with the following risk factors models (they both include the same factors but with different regression coefficients):

logit(Parasite1)=α_1,0_ + α_1,1_F_1_ + α_1,2_F_2_ + α_1,3_F_3_ + α_1,4_F_4_ + α_1,5_F_2_F_3_

logit(Parasite2)= α_2,0_ + α_2,1_F_1_ + α_2,2_F_2_ + α_2,3_F_3_ + α_2,4_F_4_ + α_2,5_F_2_F_3_

In R, the common model is summarized by: **F1+F2*F3+F4**, with F2*F3 corresponding to F2+F3+F2:F3.

**To run the example step-by-step, please process as follows:**

1. Open the R software and “Chi2corr.R” (File S2): open the script from R or copy and paste the code in a new script.
2. Run the script: select all and execute. Follow the instructions as shown below.
   1. Choose your working directory


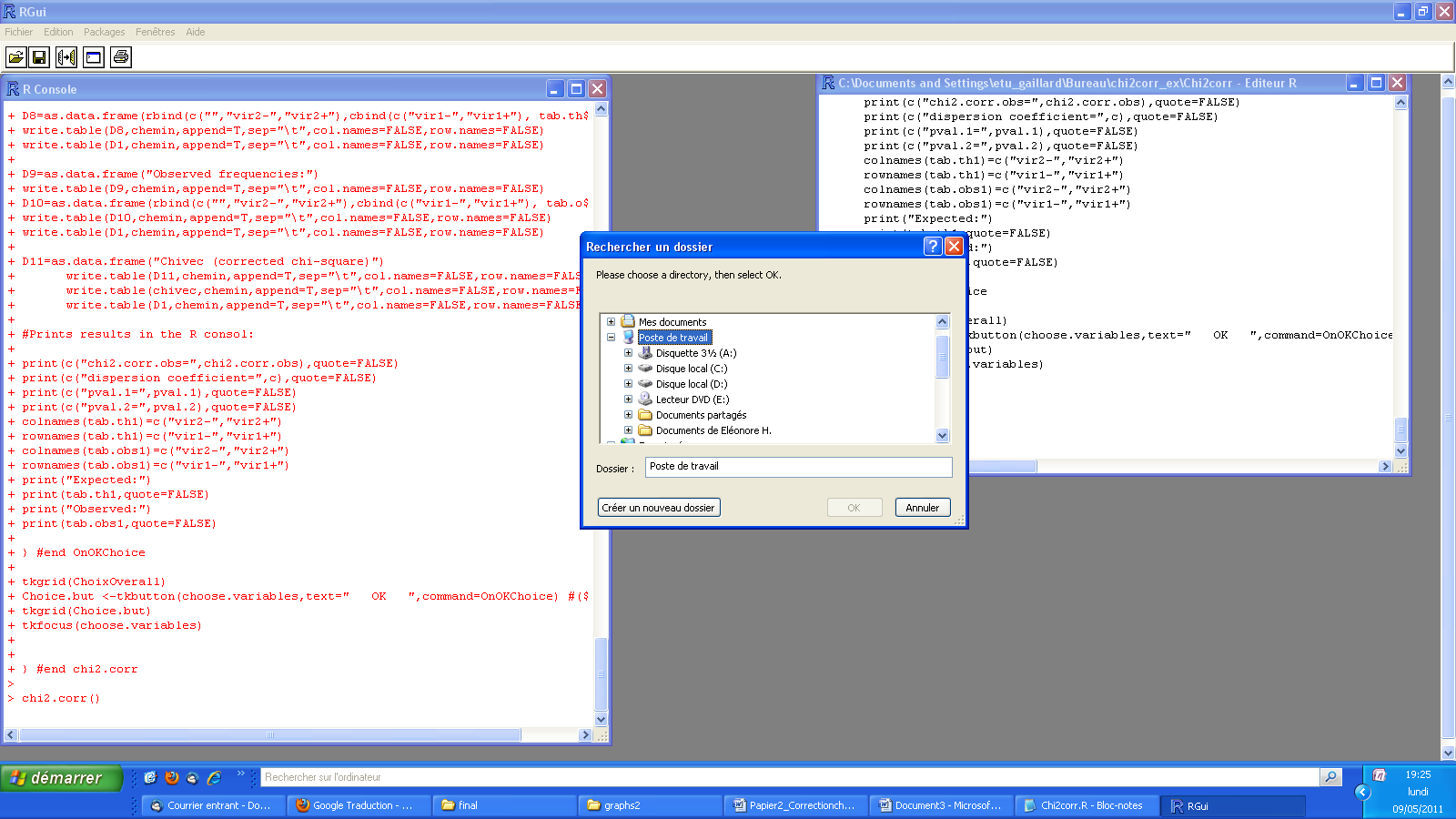

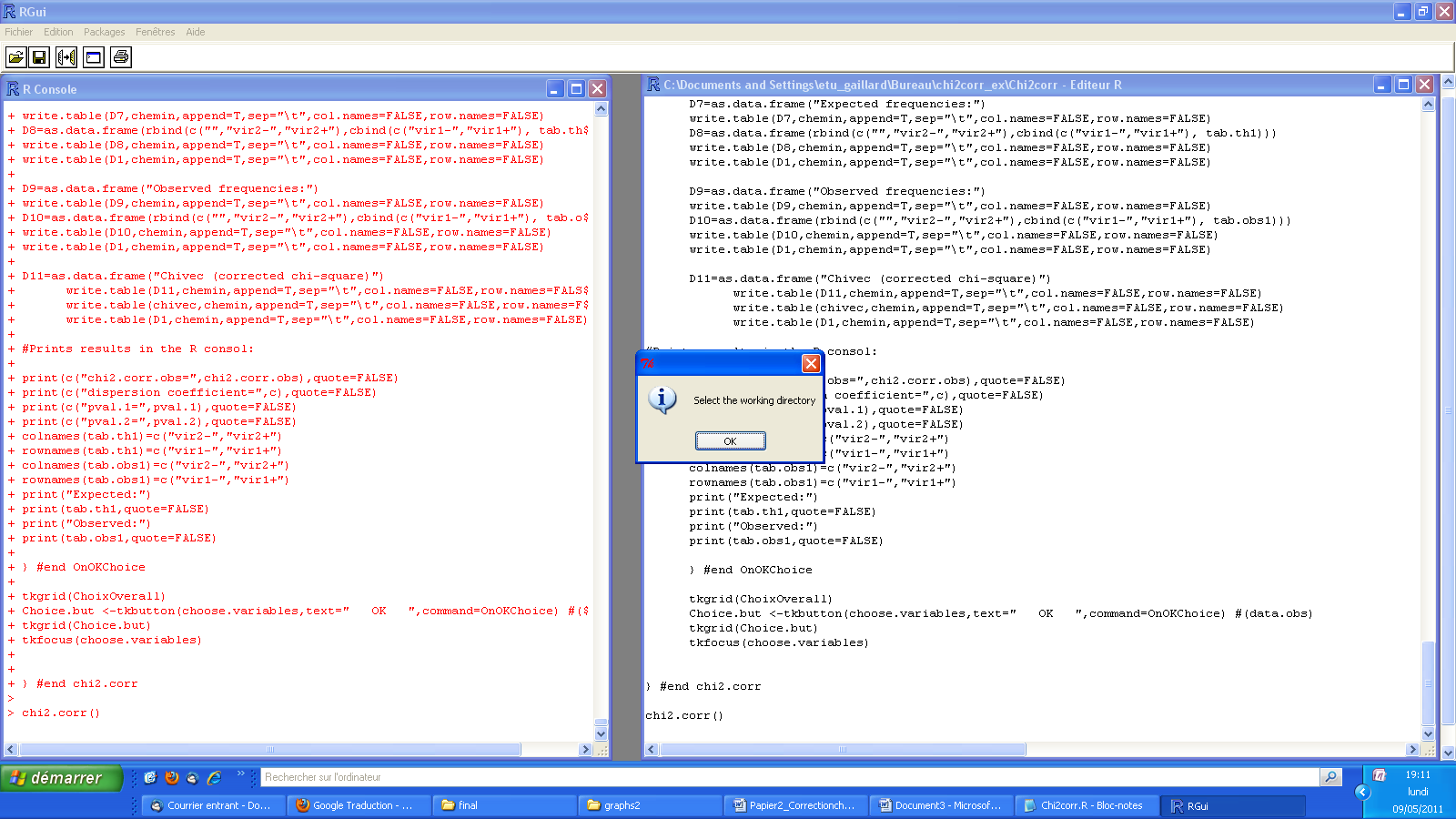


*Press the “OK” button, the selection window appears:*

*Select your working directory*

- 1. Select your data file (.txt)


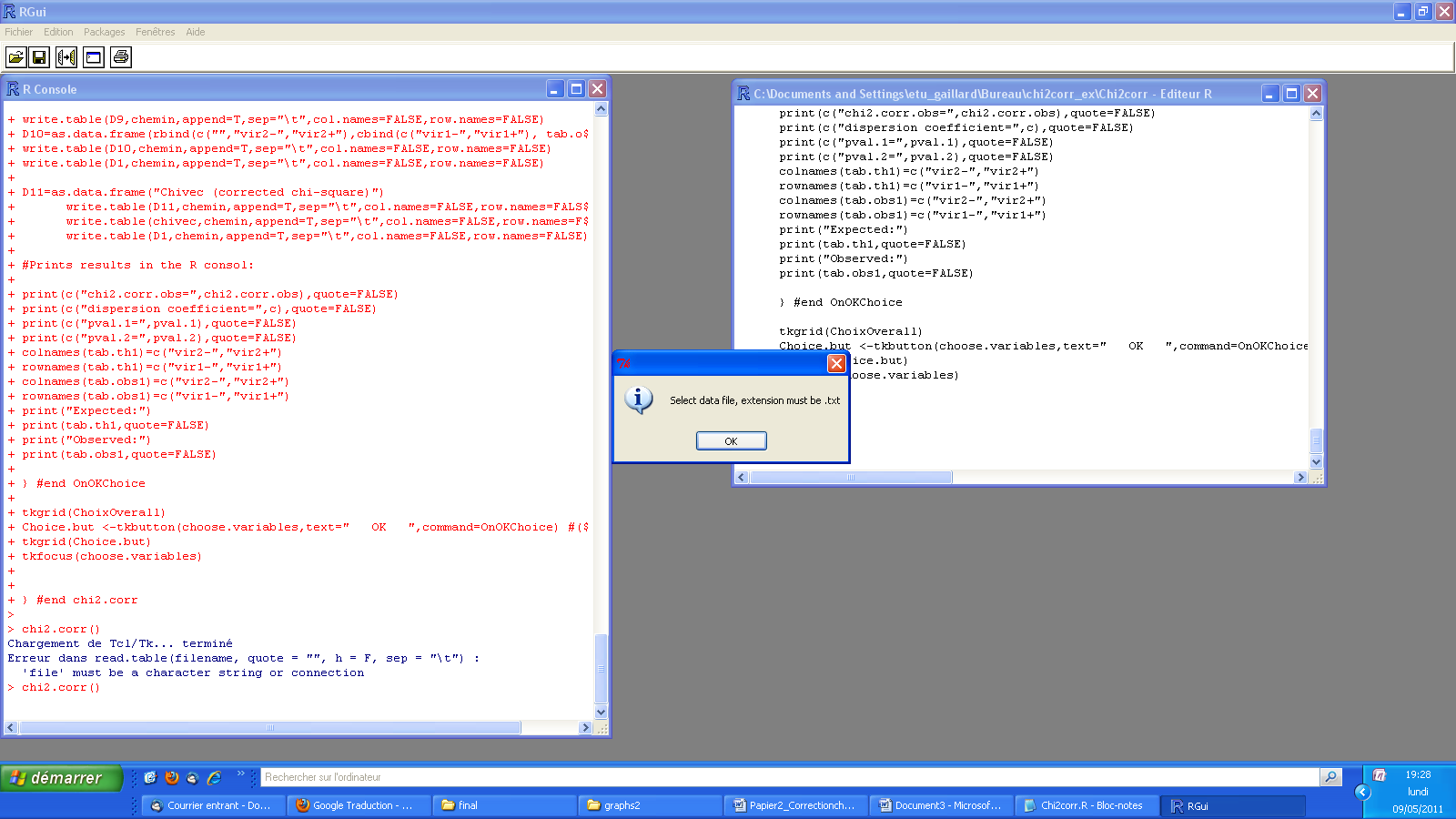


*Press the “OK” button (note the extension type reminder)*


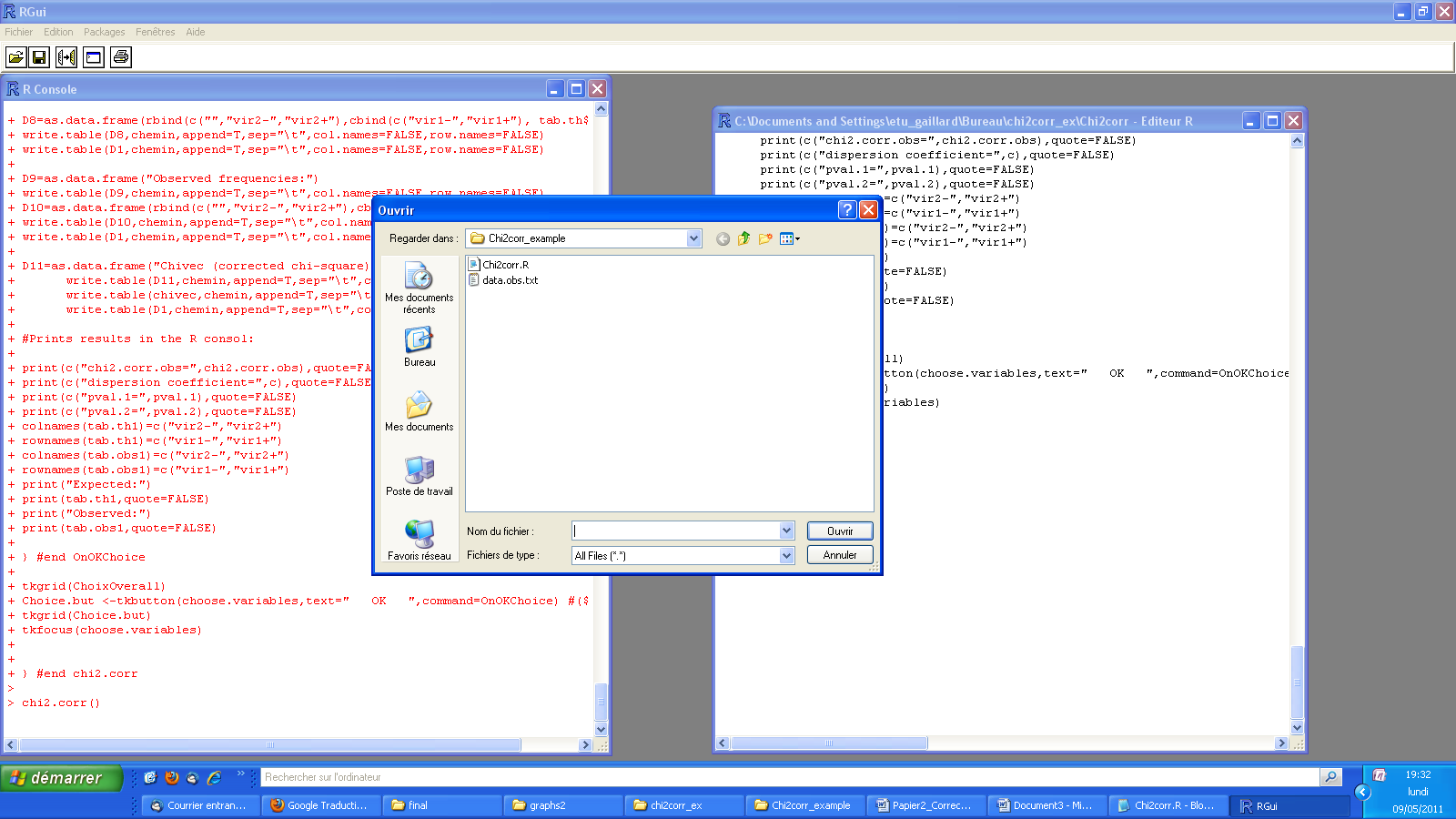


*Select your data file. In this example, select: “SupplementaryFile4_data_example.txt”*

If you select a file with another extension, an error message will appear and you will need to restart by executing chi2.corr again.


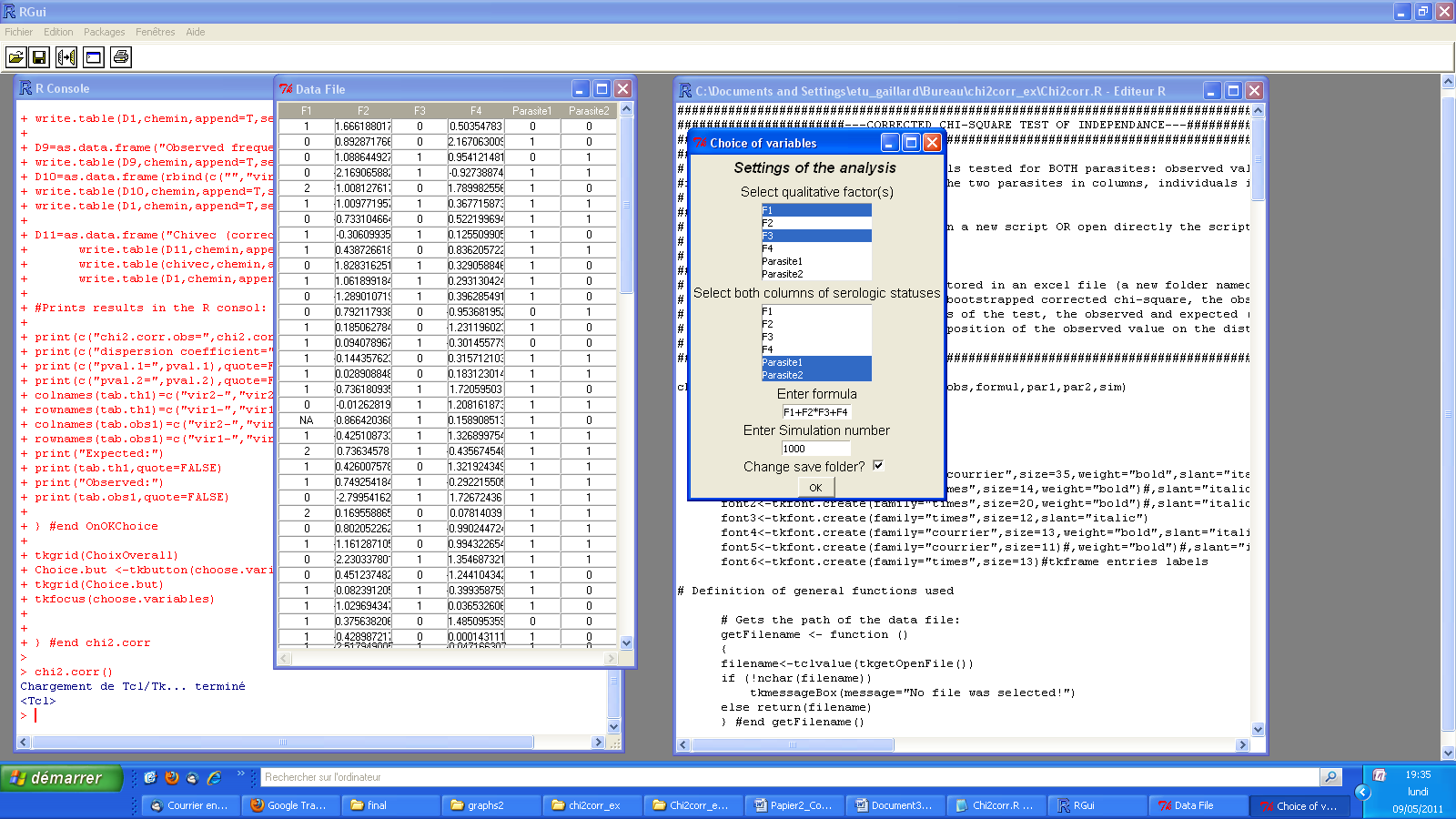


- 1. Give the required information:

Highlight columns corresponding to the qualitative factors and those correspon-ding to the serological statuses.

The number of wanted bootstraps

Tick only if you want to save results in another folder than the chosen working directory; if ticked you’ll access a new selection window.

The risk factors model (e.g. the simplest model including all risk factors common to both parasites). Note that F1*F2 corresponds to F1+F2+F1:F2.

- 1. The parametric bootstrap will run once you have pressed the “OK” button.
  2. Outcomes:

*2.5.1. In the R consol:*

[1] chi2.corr.obs= 0.00637 #the observed value of the corrected chi-square

[1] dispersion coefficient= 0.64409

#Pvalue considering chi2corr is proportional to a chi2 with 1 df

[1] pval.1= 0.92078

#Pvalue obtained from the bootstrapped chi2corr

[1] pval.2= 0.929

[1] "Expected:"

vir2- vir2+

# the expected frequencies under the independence hypothesis,

# considering the risk factors.

vir1- 14.18 15.82

vir1+ 34.82 29.18

[1] "Observed:"

vir2- vir2+

vir1- 14 16

vir1+ 35 29

*2.5.2. In the “Results” folder automatically created in your working directory:*

Results are saved in an excel file named according to the form “Sim_year-month-day.xls “.

| SYNTHESIS OF RESULTS | |  |
| --- | --- | --- |
|  |  |  |
| Observed corrected chi-square | 0.00637 |  |
| Dispersion coefficient | 0.64409 |  |
| Pvalue1 | 0.92078 |  |
| Pvalue2 | 0.929 |  |
|  |  |  |
| Expected frequencies: |  |  |
|  | vir2- | vir2+ |
| vir1- | 14.18 | 15.82 |
| vir1+ 34.82 | | 29.18 |
|  |  |  |
| Observed frequencies: |  |  |
|  | vir2- | vir2+ |
| vir1- | 14 | 16 |
| vir1 35 | | 29 |
|  |  |  |
| Chivec (corrected chi-square) |  |  |
| 0.309338501918585 |  |  |
| 1.33632856884273 |  |  |
| 0.235151426038125 | | |
| 0.260200805072199 | |  |
| 0.545825054760801 | |  |
| 0.000993413678995098  **[...]** | |  |

The corrected chi-square distribution and the position of the observed corrected chi-square is drawn and also saved in the “Results” folder. The value of the observed corrected chi-square is indicated by a red star (here chi2corr.obs= 0.00637):

Note that in this example the test is not significant and the observed corrected chi-square is in the distribution. The conclusion is therefore that the proportion of double infected can be explained without invoking an interaction between the two parasites. It was expected as serological data were generated with all individuals having an independent 0.5 probability of being seropositive for each pathogen.
